# Long-term moderate treadmill exercise promotes stress-coping strategies in male and female rats

**DOI:** 10.1038/srep16166

**Published:** 2015-11-05

**Authors:** Jaume F. Lalanza, Sandra Sanchez-Roige, Igor Cigarroa, Humberto Gagliano, Silvia Fuentes, Antonio Armario, Lluís Capdevila, Rosa M. Escorihuela

**Affiliations:** 1 Institut de Neurociències, Departament de Psiquiatria i Medicina Legal, Fac de Medicina, Universitat Autònoma de Barcelona, 08193 Bellaterra, Barcelona, Catalonia, Spain; 2 Laboratori de Psicologia de l’Esport, Departament de Psicologia Bàsica, Fac Psicologia, Universitat Autònoma de Barcelona, 08193 Bellaterra, Barcelona, Spain; 3 Carrera de Kinesiología, Facultad de Salud, Universidad Santo Tomás, Los Ángeles, región del Bio-Bio, Chile; 4 Red de trastornos adictivos (RTA) and Institut de Neurociències, Unitat de Fisiologia Animal, Universitat Autònoma de Barcelona, 08193 Bellaterra, Barcelona, Spain

## Abstract

Recent evidence has revealed the impact of exercise in alleviating anxiety and mood disorders; however, the exercise protocol that exerts such benefit is far from known. The current study was aimed to assess the effects of long-term moderate exercise on behavioural coping strategies (active vs. passive) and Hypothalamic-Pituitary-Adrenal response in rats. Sprague-Dawley male and female rats were exposed to 32-weeks of treadmill exercise and then tested for two-way active avoidance learning (shuttle-box). Two groups were used as controls: a non-handled sedentary group, receiving no manipulation, and a control group exposed to a stationary treadmill. Female rats displayed shorter escape responses and higher number of avoidance responses, reaching criterion for performance earlier than male rats. In both sexes, exercise shortened escape latencies, increased the total number of avoidances and diminished the number of trials needed to reach criterion for performance. Those effects were greater during acquisition in female rats, but remained over the shuttle-box sessions in treadmill trained male rats. In females, exercise did not change ACTH and corticosterone levels after shuttle-box acquisition. Collectively, treadmill exercise improved active coping strategies in a sex-dependent manner. In a broader context, moderate exercise could serve as a therapeutic intervention for anxiety and mood disorders.

An active lifestyle has been extensively shown to be an effective way to promote physiological and mental health benefits. The guidelines that health professionals are using recommend at least 150 min of weekly moderate-intensity aerobic activity to promote health benefits^1^,^2^,^3^. Depending on the body weight, 150 min of moderate-intensity aerobic activity per week is equivalent to 800–1200 Kcal^4^. This dose of activity has been associated with favourable changes in a wide range of health parameters such as blood pressure, lipid and lipoprotein profiles, markers of inflammation, cognitive function, mental quality of life or depression in addition to other parameters^4^,^5^,^6^,^7^,^8^. More recently, exercise has emerged as a promising intervention for alleviating anxiety-like behaviour^9^ (for a review). For example, moderate-intensity exercise (i.e. 150–180 min/week, for 8–12 weeks) has been shown to improve symptoms of agoraphobia, panic disorder or generalized anxiety disorder^9^. In individuals without a mental disorder, both short (i.e. 1–3 sessions) and chronic (i.e. 8–12 weeks) interventions of moderate-intensity exercise reduced trait and state anxiety, and diminished anxiety sensitivity or mood^9^.

Using animal models, there are two main categories for measuring anxiety: unconditioned response tests (which require no training and usually have eco/ethological validity) and conditioned response tests (which often require training and involve learned/punished responses)^10^,^11^. Unconditioned tests, such as the elevated plus-maze and open field tests, evaluate coping strategies under novel threatening conditions (i.e. environments) that approximate to the natural open or unprotected spaces, shown to elicit anxiety^10^. Conversely, conditioned tests evaluate coping strategies by pairing a neutral stimulus with a threatening stimulus (electric shock) to promote specific avoidance/escape or defensive behavioural responses. For example, the animal may use passive or active responses to escape or avoid the shock^11^.

However, the rodent literature studying the effects of exercise on anxiety-like behaviour is controversial. On one hand, protocols of vigorous-intensity exercise (20 m/min, 45 min/day, 5 days/week for 18 weeks) did not change anxiety-like behaviour as measured in the elevated plus-maze, the open field, social interaction and conditioned freezing^12^. On the other hand, other studies have reported that treadmill exercise protocols of similar intensity or middle-intensity (14 m/min, 60 min/day) but longer duration (24, 64 weeks) led to a reduction of anxiety-like behaviour in the elevated plus-maze and the open field tests^13^,^14^, and counteracted the impairments produced by postnatal-maternal deprivation on fear memories^15^. In a more recent study, four weeks of treadmill exercise (30–60 min/day, 10–15 m/min, 5 days/week) diminished anxiety-like behaviour as measured in an open field in a rat model of Alzheimer’s disease^16^. In addition to the behavioural changes, long-term moderate-intensity treadmill training (36 weeks, 12 m/min, 30 min, 4–5 days/week) also reduced the hormonal response to acute mild and severe stress^17^. Furthermore, models of voluntary exercise (i.e. wheel running for 6 months) have shown to reverse the behavioural helplessness escape deficits induced by uncontrollable foot shock stress^18^. Altogether, exercise practise seems to decrease anxiety-like behaviour when administered under certain protocol conditions.

Here we aimed to investigate the impact of long-term moderate-intensity treadmill exercise on coping strategies in a conditioned conflict test involving anxiety. To test this hypothesis, we used the two-way (shuttle-box) active avoidance paradigm, which involves a fear-mediated conflict between a tendency to freeze against a tendency to actively escape (i.e. “passive/active avoidance conflict”) in a box (shuttle-box) with two compartments separated by an open door. In order to escape or avoid the upcoming foot shock, signalled by a light and/or a tone, the animal must learn to change between the two compartments. Low baseline levels of anxiety (i.e. in low anxious rats^19^, or via administration of anxiolytic drugs^20^,^21^, or early postnatal handling^19^) are associated with enhanced performance and faster active avoidance responses in this task, whereas high anxious profiles (i.e. in anxious rats, or induced by administration of anxiogenic drugs or previous stress challenges) increase the freezing response and favour passive avoidance, resulting in longer response latencies and slower acquisition^19^,^20^,^21^.

From multiple lines of evidence using animal and human subjects, responses to fear, anxiety responses, coping strategies and the putative mechanisms underlying those emotional processes^22^,^23^,^24^,^25^,^26^ have been shown to be sex-specific. Prevalence of anxiety disorders is 60% higher in women than in men^27^, which also show different avoidance behaviours^28^ and treatment responses^29^,^30^. Hence, we aimed to study the impact of treadmill exercise (32-weeks) on active avoidance behaviour in male and female rats, to account for sex differences. Additionally, we previously found that treadmill training diminished ACTH stress response in male rats, both immediately after one session of shuttle-box and again 30 minutes after its termination^17^. In the present study we aimed to investigate whether hormonal stress response was modified after shuttle-box acquisition in treadmill trained female rats. To this aim plasma levels of ACTH and corticosterone were measured under resting conditions and after shuttle-box acquisition. The main advantages of our model over other models of exercise are that a) using a treadmill model, the duration and intensity of exercise can be adjusted by the experimenter, and hence control for the heterogeneity described with other models (e.g. voluntary exercise training^31^); and b) including two control groups: a sedentary male (M) and female (F) group, receiving no manipulation (M-SED and F-SED, respectively), and a control male and female group (M-CON and F-CON, respectively) exposed to a stationary treadmill under the same conditions as for the treadmill (TM) group can serve to detect the influence of other variables, such as daily handling and mere exposure to the treadmill apparatus^32^.

## Results

### Sex and treadmill exercise effects on body weight gain

Over the treadmill training, male rats (M-SED: 201.7 ± 20.0 g; M-CON: 203.2 ± 15.9 g; M-TM: 181.2 ± 16.8 g) gained more weight than female rats (F-SED: 110.4 ± 14.6 g; F-CON: 85.4 ± 11.0 g; F-TM: 113.6 ± 11.4 g; sex effect: F(1, 63) = 47.62, p = 0.001), but treadmill exercise did not affect body weight gain (p = 0.753). When the initial body weight (i.e. at the start of the shuttle-box experiment) was included in the analysis as a covariant, the observed sex differences disappeared (F(1, 63) = 49.30, p = 0.001; sex: p = 0.715; exercise: p = 0.612), suggesting that initial body weight differences between male and female rats may underlie the apparent sex-dependent effects of treadmill exercise. *Post-hoc* pair comparisons revealed that these sex-dependent effects were significant in male and female groups of sedentary and control conditions (M-SED vs. F-SED p = 0.002, M-CON vs F-CON: p = 0.001), but not between the M-TM and F-TM groups (p = 0.088). Moreover, F-CON and F-TM groups residually differed in body weight gain (p = 0.07, Student’s t-test), F-TM group showing higher body weight than F-CON at the end of the treadmill intervention.

### Sex differences on two-way active avoidance performance

Female rats performed more crossings than male rats during the habituation period (sex: F(1, 63) = 15.24, p = 0.001; Table 1).

Overall female rats showed shorter escape latencies (Kruskall-Wallis: χ^2^(5) = 24.03, p = 0.001), more avoidance responses (or greater number of escapes; χ^2^(5) = 16.13, p = 0.006) and reached criterion for performance earlier (sex: F(1, 63) = 9.90, p = 0.003), than male rats (Table 1). *Post-hoc* comparisons revealed that each female group showed a mean escape latency shorter than the one shown by the corresponding male group receiving the same type of intervention (SED: p = 0.005, CON: p = 0.035 or TM: p = 0.046; Table 1).

With regard to avoidance responses, the F-SED group performed more avoidance responses than the M-SED group (p = 0.047), but there were no differences between the other groups on this variable (Table 1).

### Treadmill exercise effects on two-way active avoidance performance

Table 1 shows the effects of treadmill exercise on shuttle-box performance. Treadmill exercise showed a tendency to affect the number of crossings during the habituation period in an opposite manner for male and female rats, i.e. increasing the number of crossings in male rats but decreasing in female rats (‘sex × exercise’: F(2, 63) = 3.12, p = 0.052). *Post-hoc* analysis revealed that both M-SED and M-CON groups showed lower number of habituation crossings than the corresponding F-SED (p = 0.027) and F-CON groups (p = 0.021), whereas the M-TM and F-TM groups did not differ, both performing 10 crossings during the habituation period.

Treadmill exercise diminished the number of trials needed to achieve the criterion for performance (exercise: F(2, 63) = 7.27, p = 0.002), the F-TM group achieving performance criteria during the first session (trials 1–30), the F-CON, the F-SED and M-TM groups during the second session (trials 31–60), and the M-SED and M-CON groups during the third session (trials 61–90). Treadmill exercise improvements on this variable appeared to be greater in female rats compared with male rats, since the F-TM group needed fewer trials than the F-SED group (p = 0.002), whereas the corresponding M-TM and M-SED groups differed at a lower significant level (p = 0.036).

Significant differences on mean escape latencies and total avoidance responses appeared in the analysis (χ^2^(5) = 24.03, p = 0.001, and χ^2^(5) = 16.13, p = 0.006, respectively). *Post-hoc* comparisons revealed that the M-TM group displayed faster escape latencies than the M-SED group (p = 0.001), whereas F-TM and F-SED groups showed similar mean escape latencies (p = 0.08). Moreover, both the F-TM and M-TM groups showed greater total number of avoidance responses compared with the corresponding F-SED (p = 0.025) or M-SED (p = 0.01) groups.

### Different sex and treadmill exercise response patterns on the transition from escape/avoidance behaviour

**Figure 1:**
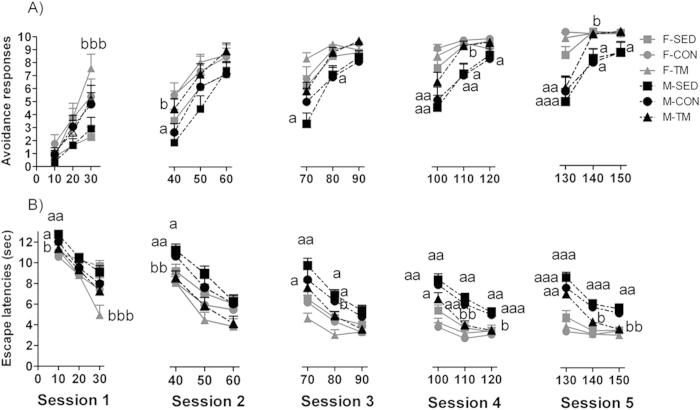
Mean ± SEM of avoidance responses and escape latencies in the shuttle-box over three 10-trial blocks of sessions 1–5 for sedentary (SED), control (CON) and treadmill trained (TM) male (M) and female (F) rats. ^a^*p* < 0.05, ^aa^p < 0.01 and ^aaa^p < 0.001 vs. the corresponding female group (same intervention type); ^b^p < 0.05, ^bb^p < 0.01 and ^bbb^p < 0.001 vs. the corresponding SED group (same sex, after decomposition of significant interactions).

The number of avoidance responses and escape latencies over the three 10-trial blocks of sessions 1–5 are depicted in Fig. 1A,B. As expected, all the animals improved overall performance over trials (avoidance responses: F(2, 116) = 199.6, p = 0.001; escape latencies: F(2, 116) = 295.2, p = 0.001) and over sessions (avoidance responses: F(4, 232) = 194.4, p = 0.001; escape latencies: F(4, 116) = 168.3, p = 0.001). A significant ‘block × session’ interaction appeared in the analysis (avoidance responses: F(8, 464) = 6.6, p = 0.001; escape latencies: F(8, 464) = 9.3, p = 0.001), suggesting that all the rats improved their performance during the final 10-trials of each session.

The patterns of avoidance responses and escape latencies were different between male and female rats over blocks and/or sessions. A significant ‘block × sex’ (avoidance responses: F(2, 116) = 14.86, p = 0.000; escape latencies: F(2, 116) = 19.00, p = 0.001), ‘session × sex’ (avoidance responses: F(4, 232) = 2.91, p = 0.043; escape latencies: F(4, 232) = 6.23, p = 0.001), and ‘block × session × sex’ interactions (avoidance responses: F(8, 464) = 3.59, p = 0.002; escape latencies: F(8, 464) = 2.26, p = 0.003; Fig. 1A,B) revealed that overall female rats showed increased avoidance responses and diminished escape latencies than male rats. In fact, the F-TM group showed the best initial performance as indicated by the scores of 7 avoidance responses and mean escape latency shorter than 5 sec that they showed in the third 10-trial block of session 1. From session 2 onwards, the effect of treadmill training in the F-TM group was abolished (Fig. 1A,B).

The repeated measures analysis over blocks and sessions also revealed a significant ‘block × session × exercise’ interaction for avoidance responses (F(16, 464) = 2.98, p = 0.001; Fig. 1A), attributable to a higher number of avoidance responses in M-TM rats in comparison to M-SED rats during the first block of session 2, and during the second block of sessions 4 and 5 (Fig. 1A). With regard to escape latencies, the significant ‘block × session × exercise’ (F(16, 464) = 2.53, p = 0.002) and ‘block × session × sex × exercise’ interactions (F(16, 464) = 1.75, p = 0.046; Fig. 1B) revealed that: i) the F-TM group performed shorter escape latencies than the F-SED group in the third 10-trial block of session 1 (p = 0.001), whereas that difference was not observed among the corresponding M-TM and M-SED groups; ii) the F-TM group responded with escape latencies below 6 s from the first block of session 3 and stabilized around 4 s over the second and third block of that session, whereas the other groups required more trials to stabilize escape latencies over blocks and/or sessions; iii) conversely, the M-TM group showed shorter escape latencies than the M-SED group in blocks 1 (p = 0.009), 2 (p = 0.013) of session 2, block 2 (p = 0.039) of session 3, and blocks 2–3 of sessions 4 (p = 0.024 and p = 0.007) and 5 (p = 0.043 and p = 0.008); iv) both the M-SED and M-CON groups showed longer escape latencies than the M-TM group.

**Figure 2:**
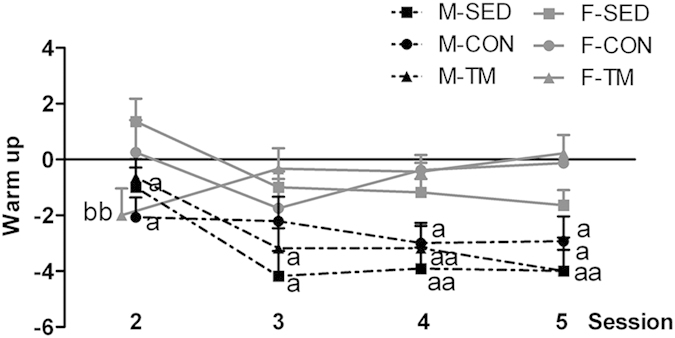
Mean ± SEM of warm up scores for sedentary (SED), control (CON) and treadmill trained (TM) male (M) and female (F) rats. Warm up index indicates the drop-off in avoidance behaviour from one session to the next. Warm up was less pronounced in female rats compared with male rats. ^a^*p* < 0.05 and ^aa^p < 0.01 vs. the corresponding female group (same intervention type); ^bb^p < 0.01 vs. the corresponding SED group (same sex, after decomposition of significant interactions.

As for the warm up process, a closer inspection of Fig. 2 indicates that the warm up scores were overall higher in male than female rats (sex: F(1, 58) =  22.03, p = 0.001). The warm up scores changed significantly from session 2 to session 3 and then stabilized in a difference of 0–2 avoidance responses in female rats and 3–4 avoidance responses in male rats (‘session × sex’: F(3, 174) = 2.96, p = 0.003). Moreover, the progression of the warm up scores between the F-TM and M-TM groups and their respective SED and CON groups was significantly different (‘session × sex × exercise’: F(6, 174) = 3.094, p = 0.007). Precisely, in female rats the F-TM group performed 8 avoidance responses during the last block of session 1, 5.6 avoidances in the first block of session 2, and then stabilized around a warm up score of 0. The F-CON group reached a warm up score of 0 in session 5, whereas the F-SED group showed a negative warm up score of −2 in session 5. Conversely, all groups of male rats increased negative warm up scores from session 2 onwards and stabilized around a −4 score.

The number of inter trial crossings performed over sessions 1–5 are shown in Table 2. Overall, the number of inter trial crossings during task acquisition changed over the sessions (F(4, 232) = 3.991, p = 0.004), but no significant session × sex (F(4, 232) = 1.103, p = 0.461), session × exercise (F(8, 232) = 1.745, p = 0.107) and session × sex × exercise interactions (F(8, 232) = 1.154, p = 0.328) appeared in the two-way ANOVA analysis for repeated measures. However, significant sex differences emerged in the analysis (F(1, 58) = 20.019, p = 0.001), indicating that female rats performed more inter trial crossings than male rats. Treadmill training showed an overall tendency to increase inter trial crossings (F(2, 58) = 2.523, p = 0.089).

### Hormonal response

**Figure 3:**
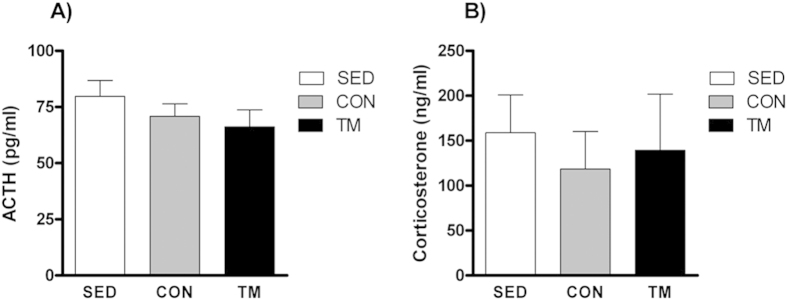
Morning levels of ACTH (A) and corticosterone (B) in sedentary (SED), control (CON) and treadmill (TM) female rats under resting conditions did not differ (n = 8–10/group).

**Figure 4:**
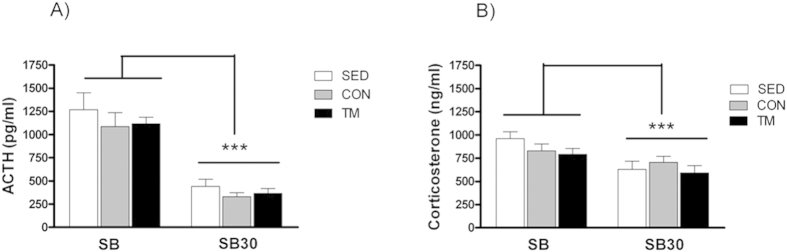
Lower ACTH (A) and corticosterone (B) levels were found 30 min after shuttle-box acquisition termination (SB30) in all female rats compared with the hormone levels immediately after the shuttle-box session. Means ± SEM are shown. ***p < 0.05 (overall time effect).

The analysis of morning hormones showed no differences among female groups for ACTH (F(2, 27) = 1.054, ns; Fig. 3A) or corticosterone (F(2, 27) = 0.166, ns; Fig. 3B) plasma levels under resting conditions. 30 min after shuttle-box performance, ACTH (F(1, 25) = 135.28; p = 0.001; Fig. 4A) and corticosterone (F(2, 25) = 17.63; p = 0.001; Fig. 4B) plasma levels were lower in comparison to plasma levels measured immediate after shuttle-box acquisition. There were no group differences for ACTH and corticosterone plasma levels immediately after shuttle-box acquisition (F(2, 27) < 1.660, ns) or after 30 min of recovery (F(2, 27) < 0.771, ns).

## Discussion

Evidence from human studies indicates that exercise might be an effective intervention for anxiety disorders^9^. Here we showed that treadmill exercise improved active avoidance performance in a conditioned animal model of anxiety. The exercise benefit is sex-specific, according to the pattern of performance shown by male and female rats. We also showed sex differences on shuttle-box performance, female rats acquiring active avoidance behaviour faster and earlier than did male rats. They reached higher levels of performance than male rats as indicated by shorter escape latencies, higher number of avoidance responses, fewer trials needed to reach the performance/learning criterion of eight consecutive avoidances and lower warm up scores.

To our knowledge, the current results demonstrate for the first time that long-term moderate treadmill exercise improved two-way active avoidance behaviour in both male and female rats, thus resulting in better conflict coping strategy. Such an observation is in keeping with previous studies showing reduced anxiety-like behaviour in unconditioned tests of anxiety^13^,^14^,^33^,^34^ and reduced ACTH response to mild and severe stressors following treadmill exercise^17^, but is in disagreement with another study showing lack of treadmill effects on fear conditioning or escape-learning deficit produced by uncontrollable stress^35^. The apparent discrepancies on shuttle-box performance suggest that the effects of treadmill exercise may vary as a function of the protocol of exercise used (ours, low intensity and chronic exposure vs. higher intensity and shorter exposure in the later^35^), but subtle differences regarding elicitation of fear versus anxiety among the various situations could contribute to the discrepancies.

The effects of treadmill exercise appeared at different time points, during acquisition in female rats, and later on, from session 2 onwards in male rats. F-TM rats improved avoidance responses and escape latencies, and reached criterion for performance in the third block of session 1. M-TM rats improved the total number of avoidance responses in session 3 (24.64 ± 0.92, see Supplemental Table 1), maintained a similar performance in sessions 4–5, and diminished the rate of escape latencies throughout the shuttle-box testing period (see Fig. 1B). This effect is unlikely to be due to increased levels of motor activity induced by treadmill exercise, as F-TM rats did not show higher number of crossings than F-CON or F-SED rats during habituation or in the inter-trials in session 1. Similarly, in M-TM rats the numbers of crossings were not higher than M-CON or M-SED rats from session 2 onwards. It appears that in male rats, treadmill exercise may have diminished behavioural inhibition, thus promoting increased activity and facilitating active avoidance performance. This is in line with a previous study showing that long-term wheel running exercise reduced conditioned freezing and reversed the escape deficit induced by uncontrollable foot shock^18^. However that hypothesis needs to be further evaluated, since moderate treadmill exercise did not affect anxiety-like behaviour in the hole board, the elevated plus maze or the open field tests^17^, thus suggesting that anti-anxiety benefits of treadmill exercise may appear in more demanding situations than the former unconditioned tests.

The improvement in avoidance behaviour and criterion for performance observed in F-TM rats during the first session of the two-way active avoidance in comparison to F-SED and F-CON groups was not observed in subsequent sessions, all female groups reaching 27–29 avoidance responses by the 4rth session (see Supplemental Table 1). Nevertheless, it is likely that female rats were performing at ceiling, thereby masking possible differences between groups. Further experiments increasing the demands of the task may help explain this hypothesis. For instance, shorter inter-trial intervals (15 s vs. our 40 s), and/or a shorter time CS duration (5 s vs. our 10 s) have been previously shown to retard avoidance acquisition^36^. It should be noted, however, that the high exposure to handling procedures in both CON and TM rats could underlie some of the benefits observed in the CON rats (see Fig. 1). Other studies have previously shown that exposure to handling improved shuttle-box learning acquisition and diminished anxiety-like behaviour^36^,^37^,^38^,^39^. In our study, CON and TM groups showed similar levels of performance (>4.5 avoidance responses) during the third block of session 1 in comparison with the sedentary group (<3 avoidance responses; see Fig. 1A,B), who showed the slowest rates of acquisition.

Alongside the TM effects on shuttle-box performance, treadmill exercise did not modify the body weight, a result which is consistent with some studies^34^,^40^, but not others^41^. Collectively, it is possible that different types of activity promote different health outcomes^3^, which may explain the lack of changes in body weight following our treadmill training (i.e. moderate intensity, 150 min/week), but not others (i.e. higher intensity of training or duration^41^,^42^). Additionally, TM female rats slightly gained more weight than CON female rats, suggesting that exercise may increase muscle mass, as others have found for muscle weight to body weight relation mass^43^ and/or by an increase in bone mass^44^.

In regard to basal HPA hormones, the analysis showed that TM female rats did not differ from the SED and CON female groups in plasma ACTH and corticosterone levels, consistent with previous studies in our laboratory among the SED, CON and TM male groups^17^. Moreover, ACTH and corticosterone responses to shuttle-box acquisition were not modified in female treadmill trained rats. This finding is in disagreement with our previous study^17^, where treadmill trained male rats showed a reduced ACTH response after an escape-avoidance task performed in a shuttle box compared to SED and CON male rats. In that study, there were no differences in the number of avoidances or number of shocks received during acquisition among male groups, whereas in the current one, TM female rats were the only group that reached criterion for performance in session 1. The possibility that a more severe stress challenge than the two-way (shuttle-box) active-avoidance acquisition *per se* were needed to observe the benefits of exercise on HPA response to stress is unlikely, as the protective effects of exercise are better observed with lower intensity stressors and/or demanding tasks^45^. It is possible that female rats are more resistant than male rats to the beneficial effects of exercise on HPA responsiveness to stressors, in contrast to the behavioural consequences. There is evidence indicating that, female rodents tolerate higher intensities of forced exercise and are more active in the voluntary wheel running than male rodents^46^,^47^. In the current experiment all animals were trained at 12 m/min, 30 min/day, 5 days/week, which is considered as moderate exercise intensity (60–70% max.VO_2_), comparable to a brisk walking pace^14^,^48^. Thus, the present results do not allow to rule out that higher exercise ‘doses’ were needed for female rats to reduce HPA response to stress.

A second new finding of the current experiment regarding sex differences is that female rats showed less warm up than males. This is consistent with other studies showing greater acquisition of escape/avoidance behaviour in animals with less warm up scores. For instance, warm up is highly involved in the psychogenetically selection of Roman High Avoidance/Verh (RHA/Verh) and Roman Low Avoidance (RLA/Verh) rat strains for good vs. poor shuttle-box avoidance performance, the RHA/Verh strain showing greater avoidance performance and less warm up scores than RLA/Verh^19^. Moreover, the Wistar-Kyoto rats showed lower warm up scores and increased avoidance performance than Sprague-Dawley rats in an escape/avoidance paradigm using lever-presses^49^. Additionally, ovariectomized and testosterone–treated female rats showed higher warm up scores and poor active avoidance performance in the shuttle-box than the corresponding control groups^50^.

The observed sex differences in the avoidance behaviour are in agreement with other studies showing that female rats generally perform better than male rats in paradigms of active avoidance, whereas male rats outperform female rats in others tests such as the classical fear-conditioning paradigm (see^23^ and^51^ for a review). Differences in basal levels of activity^50^,^51^,^52^ and emotionality^53^, or the influence of the oestrous cycle^51^ may explain the superior active avoidance acquisition herein revealed. More active animals usually learn to actively avoid the unconditioned foot shock stimulus earlier than less active animals^51^,^52^. In the current study, and consistently with others^51^,^52^, female rats learned faster and were also more active than male rats as measured by the number of crossings during the habituation. Intriguingly, female rats also performed more inter trial crossings than male rats during the two-way active avoidance acquisition (session 1), supporting that females’ behaviour is predominantly influenced by activity^54^. In regard with emotionality, others have shown that: i) male rats usually display higher levels of emotionality than female rats in other tests measuring anxiety-like behaviour^53^; and ii) high emotionality (i.e. freezing behaviour) negatively correlated with two-way avoidance performance^55^. It is possible that under stressful events, male rats show a passive avoidance tendency that interferes with active avoidance acquisition, whereas female rats may display more active escape strategies^50^,^56^. For instance, previous exposition to inescapable stress also disrupted shuttle-box performance in male, but not female, rats^56^. This hypothesis needs to be further explored, as there were no sex differences on a lever-press escape/avoidance task in Wistar-Kyoto (WKY) rats^57^, which show high stress sensitivity but better active avoidance performance than Sprague-Dawley rats^51^. Finally, the oestrous cycle can influence escape/avoidance behaviour in the shuttle-box^51^,^58^, and the fact that the stage of the oestrous cycle was not herein measured could be a potential confounding factor masking the sex differences in shuttle-box performance. Nevertheless, the overall performance within female groups was fairly homogeneous and the rates of avoidance responses are consistent with previous literature. Additionally, gonadectomy in adulthood did not modify the active avoidance (shuttle-box) behaviour of either sex^50^,^59^, which altogether suggests that the influence of the stage of the oestrous cycle may have been relatively small.

In conclusion, long-term moderate treadmill exercise has an important impact in adulthood. Treadmill exercise, but no treadmill handling, diminished the number of trials needed to achieve the criterion for performance and improved avoidance responding in both adult male and female rats. The effects appeared during session 1 in female rats and from session 2 onwards in male rats. Altogether, moderate treadmill exercise resulted in diminished anxiety-like behaviour and more effective coping strategies. These findings are of relevance for human mental health, and may reinforce the therapeutic use of moderate exercise for improving coping strategies in the treatment of anxiety-like disorders.

## Methods

### Animals

Male and female (n = 37 and 29 respectively, 12–14 weeks of age) Sprague-Dawley rats were obtained from the *Servei d’Estabulari, Universitat Autònoma de Barcelona*. They were housed 2 per cage in standard macrolon cages (40 cm in length × 23 cm in width × 18 cm in depth) and maintained under 12 h/12 h light/dark cycle (lights on 0800 h) in standard conditions of temperature (21 ± 1 °C) and humidity (50 ± 10%), with free access to food and water. Animals of each sex were randomly assigned to three groups, balancing the total body weight before starting the training sessions: sedentary male (M-SED, n = 8, 517.5 ± 12.0 g), control male (M-CON, n = 8, 545.2 ± 9.3 g), treadmill male (M-TM, n = 11, 549.9 ± 12.2 g), sedentary-female (F-SED, n = 11, 329.5 ± 6.1 g), control female (F-CON, n = 8, 312.2 ± 6.7 g) and treadmill female (F-TM, n = 9, 321.1 ± 4.8 g). The experimental protocol was approved by the Ethics Committee of the *Universitat Autònoma de Barcelona*, and was carried out following the ‘Principles of laboratory animal care’ in accordance with the European Communities Council Directive (86/609/EEC). Body weight (g) was recorded weekly until the end of the experiment. Body weight gain was calculated as the difference between the body weight measured at the last week of training and the body weight prior to the onset of treadmill training (Table 1).

### Treadmill training

The treadmill apparatus consisted of 3 parallel runways (Exer 3/6, Columbus Instruments). The training phases were based on procedures described elsewhere^17^. Briefly, at 14-weeks of age, rats were habituated to the treadmill apparatus (0 m/min) in a single 30-min session. The following day, the intensity of the exercise was gradually increased until it reached a speed of 12 metres/minute (m/min). Animals were trained in 30-min sessions, 4–5 days/week, between 9.00 h and 14.00 h, for 32 weeks.

### Shuttle Box (SB)

Each shuttle box (Panlab, S.L.) was divided into two equally sized compartments (25 cm × 25 cm × 25 cm), both sound-attenuated, connected by an opening door (8 cm wide and 10 cm high). The first session (acquisition phase, day 1) was preceded by a 5 min habituation period followed by five 30-trial sessions administered at 24-h intervals. Each trial consisted of 10 sec of conditioned stimulus (CS, light of 7 W and sound of 2400 Hz at 40 dB, presented simultaneously), immediately followed by a scrambled electric shock (unconditioned stimulus, UCS, 0.6 mA, 20 sec) administered through the metal grid floor of the box. Crossing from one side to the other compartment (active response) terminated the CS (i.e. avoidance response) or UCS presentation (i.e. escape response), and was followed by a 40-s pause (inter trial interval). Four more sessions were administered (days 2–5) with an identical procedure, with the exception of the habituation period that was not included in sessions 2–5. Shuttle-box sessions were administered 20–22 h apart. Main outcome variables were: habituation crossings, number of inter trial crossings per session (crossings made during the 40-s pause periods), the number of trials required to achieve criterion for performance (8 consecutive avoidances, “Criterion 8”, adapted from^19^), the mean escape latency (latency to cross to the other compartment from the onset of CS presentation) and the total number of active avoidance responses (crossings in the presence of CS). To analyze the transition from escape to avoidance behaviour, the number of avoidance responses and the escape latencies during blocks of 10 trials were also recorded. We also analyzed the warm up process in sessions 2–5, which refers to starting a session at a lower performance level than was performed at the end of the previous session^19^,^57^. Warm up was calculated by the difference between the number of avoidances performed during the first 10-trial block of a session and the number of avoidances performed during the third 10-trial block of the previous session [warm up index session n = (number of avoidances performed during trials 1–10 of session n) − (number of avoidance performed during trials 21–30 of session n-1)].

### Hormones

Plasma levels of ACTH and corticosterone were measured under resting conditions (morning: 9–10 am) and after the shuttle box acquisition (session 1, immediately after and 30 min after its termination). Samples were taken by tail-nick. It consisted of gently wrapping the animals with a cloth, making a 2 mm incision at the end of the tail vein and then massaging the tail while collecting, within 2 min, 300 μl of blood into ice-cold EDTA capillary tubes (Sarsted, Granollers, Spain). The two cage-mated animals were sampled simultaneously by two experimenters, in a room different from the colony room and the testing room.

Basal samples were taken five days before SB acquisition, on a day when treadmill training was not administered to prevent possible interferences with hormonal data.

### Radioimmunoassays

Plasma ACTH and corticosterone were determined by double-antibody RIAs. The ACTH RIA used 125I-tyrosil-ACTH (Amersham-Pharmacia Biotek, Cerdanyola del Vallès, Spain) as the tracer, rat synthetic ACTH (Sigma) as the standard and an antibody raised against rat ACTH kindly provided by Dr. W. Engeland (Dept. Surgery, Univ. Minnesota, Minneapolis, USA). The corticosterone RIA used 125I-corticosterone-carboximethyloxime-tyrosine-methyl ester (ICN-Biolink 2000, Barcelona, Spain), synthetic corticosterone (Sigma) as the standard, and an antibody raised in rabbits against corticosterone–carboximethyloxime-BSA kindly provided by Dr. G. Makara (Inst. Exp. Med., Budapest, Hungary). We followed the RIA protocol recommended by Dr. G. Makara (plasma corticosteroid-binding globulin was inactivated by low pH). All samples to be compared were processed in the same assay.

### Statistical Analysis

The statistical analysis was performed using the ‘Statistical Package for Social Sciences’ (SPSS, version 18.0). Body weight gain, body weight at the beginning of the shuttle-box experiment (initial body weight included in the analysis as a covariant), number of crossings during habituation period, mean inter trial crossings, and criterion for performance (log transformed to obtain homogeneity of variances, though untransformed means are shown throughout) were analysed using a two-way analysis of variance (ANOVA) with exercise and sex as the between-subject factor, followed by Bonferroni *post hoc* tests for comparisons between groups. Those variables that did not obtain homogeneity of variance following arithmetic log transformation (total number of avoidance responses, mean escape latency) were analysed by non parametric Kruskall-Wallis followed by Mann Whitney U test for comparisons between pairs of groups. A two-way repeated measures ANOVA was used to compare performance over training across the number of avoidance responses and mean escape latencies over 10-trail blocks as within-subject factors (10-trials block [3 per session] and session [5 sessions]), exercise intervention (sedentary, control and treadmill) and sex (male, female) as between-subject factors. Repeated measures ANOVA was also applied to analyze the warm up scores over sessions 2–5, with session as within-subject factors (4 sessions) and exercise intervention (sedentary, control and treadmill) and sex (male, female) as between-subject factors. When appropriate, subsequent decomposition of the interaction among factors was conducted. One-way ANOVA analysis was applied to ACTH and corticosterone morning plasma levels under resting conditions and repeated measures ANOVA in response to the SB acquisition [between-subjects factor: exercise intervention; within subjects factor: time immediately after (SB), or 30 min after its termination (SB30)] in female rats. All values are expressed as mean ± standard error for the mean (SEM). Statistical significance was set at *p* < 0.05 for all tests.

## Additional Information

**How to cite this article**: Lalanza, J. F. *et al.* Long-term moderate treadmill exercise promotes stress-coping strategies in male and female rats. *Sci. Rep.* **5**, 16166; doi: 10.1038/srep16166 (2015).

## Supplementary Material

Supplementary Information

## Figures and Tables

**Table 1:** Effects of 32 weeks of moderate treadmill training on two-way active avoidance performance (mean ± SEM).

	Group	Habituation crossings	Criterion 8	Mean escape latencies	Total avoidance responses
Females	SED	12.4 ± 0.9	59.7 ± 7	6.2 ± 0.5	100.6 ± 6.7
	CON	14.4 ± 0.8	45 ± 8.7	5.4 ± 0.3	116.8 ± 5.7
	TM	10.6 ± 1.9	28.9 ± 5.8^b^	5.0 ± 0.3	118.3 ± 2.8^b^
Males	SED	7.7 ± 0.9^a^	78.9 ± 10.1	8.2 ± 0.6^aa^	79.6 ± 6.4^a^
	CON	9.4 ± 0.7^a^	78.9 ± 12.8	7.5 ± 0.6^a^	87.6 ± 11.1
	TM	10.3 ± 0.9	48.5 ± 6.4	6.1 ± 0.3^a^	105.6 ± 4.9^b^

SED, sedentary; CON, control; TM, treadmill group (n = 8–14 per group). ^a^p < 0.05 and ^aa^p < 0.01 vs. the corresponding group of female rats (same type of intervention); ^b^p < 0.05 vs. the corresponding SED group (same sex) after significant two-way ANOVA or Kruskall-Wallis test.

**Table 2:** Inter trial crossings performed during the active avoidance sessions (mean ± SEM).

	Group	Session 1	Session 2	Session 3	Session 4	Session 5
Females	SED	7.82 ± 1.600	13.36 ± 3.571	10.27 ± 3.534	9.73 ± 2.680	6.00 ± 1.495
	CON	9.00 ± 1.822	9.88 ± 2.806	9.50 ± 4.314	8.75 ± 2.498	7.38 ± 1.700
	TM	6.11 ± 0.82	17.89 ± 3.98	21.78 ± 4.76	12.78 ± 3.38	11.78 ± 3.36
Males	SED	1.36 ± 0.24^aaa^	4.82 ± 1.10	4.64 ± 1.42	4.82 ± 1.81	3.36 ± 1.18
	CON	3.86 ± 0.69	5.14 ± 1.08	6.07 ± 1.47	4.36 ± 1.25	7.79 ± 2.74
	TM	5.27 ± 2.90	8.27 ± 3.84	7.82 ± 2.39	6.18 ± 1.58	6.64 ± 1.90

SED, sedentary; CON, control; TM, treadmill group (n = 8–14 per group). ^aaa^p < 0.001 vs. F-SED group.
