# Supplementary material for: Long-term moderate treadmill exercise promotes stress-coping strategies in male and female rats
Source: Sci Rep. 2015 Nov 5;5:16166. doi: 10.1038/srep16166 (PMC4633642; doi:10.1038/srep16166)
Supplement: Supplementary Information [file srep16166-s1.doc]

**Long-term moderate treadmill exercise promotes stress-coping strategies in male and female rats**

Jaume F Lalanza, Sandra Sanchez-Roige, Igor Cigarroa, Humberto Gagliano, Silvia Fuentes, Antonio Armario, Lluís Capdevila, Rosa M Escorihuela.

**Supplementary information:**

Supplementary Table 1. Total avoidances per sessions (mean ± se).

|  |  | Session 1 | Session 2 | Session 3 | Session 4 | Session 5 |
| --- | --- | --- | --- | --- | --- | --- |
| Females | SED | 4.6±1.8 | 17.2±2.6 | 23.8±1.9 | 27.0±0.9 | 27.9±0.6 |
|  | CON | 10.9±2.8 | 21.7±2.3 | 25.6±0.9 | 29.1±0.40 | 29.4±0.3 |
|  | TM | 12.1±1.6 | 22.6±1.3 | 27.1±0.6 | 27.6±0.6 | 29.0±0.4 |
| Males | SED | 4.9±1.5 | 14.0±2.0 | 19.1±1.5 | 20.6±1.8 | 21.0±2.1 |
|  | CON | 8.8±1.9 | 16.3±2.3 | 20.3±2.6 | 20.8±2.6 | 21.5±2.7 |
|  | TM | 8.9±2.0 | 20.9±2.1 | 24.6±0.9 | 25.8±0.9 | 25.4±0.9 |

SED, sedentary; CON, control; TM, treadmill group (n=8-14 per group).

A significant effect of session (F(4,232)=113.2, p<0.01) and a significant ‘session x exercise intervention’ interaction (F(8,232)=4.42, p<0.01) appeared in the repeated measures analysis of total avoidances (log transformed) [session as within-subject factors (5 sessions); exercise intervention (sedentary, control and treadmill) and sex (male, female) as between-subject factors]. The between-subject factors revealed significant effects of sex (F(1,58)=5.012, p=0.029) and exercise intervention (F(2,58)=3.92, p=0.025), and *post-hoc* Bonferroni tests revealed overall significant differences between SED and TM groups (p=0.024).
